# Reduction of egg reappearance period of cyathostomins in naturally infected horses after increasing doses of ivermectin in Brazil: a grim picture for sustainable parasite control

**DOI:** 10.1590/S1984-29612024043

**Published:** 2024-08-12

**Authors:** Marcelo Beltrão Molento, Julia Dall’Anese, Desiree Vera Pontarolo, Yara de Oliveira Brandão, Ursula Yaeko Yoshitani

**Affiliations:** 1 Laboratório de Parasitologia Clínica Veterinária, Departamento de Medicina Veterinária, Universidade Federal do Paraná – UFPR, Curitiba, PR, Brasil

**Keywords:** Equine, cyathostomins, horse health, infection, macrocyclic lactone, fecal egg count reduction test, Cavalo, ciatostomíneos, saúde equina, infecção, lactonas macrocíclicas, teste de redução da contagem de ovos

## Abstract

Cyathostomins are the largest group of parasites in horses that can be controlled by ivermectin (IVM). This study aimed to run a four-dose titration trial of IVM in 28 naturally infected Thoroughbred yearlings. The local Strongyle population had been recorded to be resistant to IVM (200 µg/kg). The parasite fecal egg count (FEC) was performed to investigate the egg reappearance period (ERP) of two and five weeks (w2pt and w5pt) after IVM treatment. FEC was > 1000 on day zero for all groups. Although 100% FEC reduction was reported at w2pt for all concentrations, the FEC at w5pt revealed < 83% efficacy. This study reports the reduction of ERP using the label dose as well as 300, and 400 µg/kg (double dose) of IVM. The protocol allowed IVM to significantly suppress FEC w2pt although not eliminating adult worms, failing to guarantee an extension of its protection period over 8 weeks. Moreover, the FEC at w5pt possibly means the infection was not cleared, and worms reestablished egg laying. We raised the possibility of withdrawing IVM of control programs when the drug has less than 80% FEC reduction at w5pt.

## Resumo

Ciatostomíneos são o maior grupo de parasitos de equinos, controlados com ivermectina (IVM). Este estudo teve o objetivo de realizar um teste com quatro doses de IVM, em 28 potros Puro-Sangue Inglês. Os estrôngilos eram resistentes a IVM (200 µg/kg). A contagem de ovos por grama de fezes (OPG) foi realizada após tratamento (pt), para determinar o período de reaparecimento de ovos (PRO) após duas e cinco semanas (s2pt e s5pt). A OPG foi >1000 para todos os grupos. Observou-se 100% de redução na OPG na s2pt para todas as concentrações de IVM. A OPG na s5pt revelou < 83% de redução. Este estudo relata uma redução considerável do PRO utilizando a dose de bula, e as doses de 300 e 400 μg/kg (dobro da dose) de IVM contra ciatostomíneos em equinos. O protocolo permitiu observar a redução da postura de ovos na s2pt com IVM, falhando na garantia da extensão de proteção da droga. O reaparecimento da OPG significa que a infecção não foi eliminada. Foi levantada a possibilidade de restringir o uso de IVM, quando o medicamento atingir menos de 80% de redução da OPG na s5pt.

## Introduction

Horses (*Equus ferus caballus* Linnaeus, 1758) are herbivore mammals present on every continent that harbor several parasites, i.e., cyathostomins (small strongyles), *Strongylus* spp., *Parascaris* spp., *Anoplocephala* spp., and *Dictyocaulus arnfieldi*. Cyathostomins are the largest and the most diverse group of intestinal parasites, with a high (> 90%) worldwide prevalence. These parasites cause severe episodes of diarrhea, pain, and colic in horses of all ages when present in heavy infections (Love et al., 1999), and their control is based on the regular use of anthelmintics.

Treatment strategies rely heavily on using macrocyclic lactones, such as ivermectin (IVM) in increasing doses and drug combinations. As a consequence, the adoption of suppressive treatment protocols caused parasite populations to a state of multiple anthelmintic resistance (Molento et al., 2008). The objective of such a strategy is to increase the drug spectrum by additive effect, extending the pharmacological protection of combinations and high doses (Scare et al., 2020). Even though adverse effects may happen after treatment, IVM has a high margin of safety, showing toxic signs when used at 10 times the therapeutic dose (Wescott, 1987).

Anthelmintic resistance is a worldwide problem among cyathostomin populations. The inability of dewormers to reach the intended levels of effectiveness can be measured through the fecal egg count reduction test (FECRT). The protocol considers the expected efficacy of an anthelmintic calculated from fecal egg counts (FEC) pre- and post-treatment (Coles et al., 1992). The egg reappearance period (ERP) stands for the moment (in weeks) when the egg production exceeds 10% of pre-treatment FEC levels (von Samson-Himmelstjerna et al., 2007). The ERP is a primary indicator of anthelmintic resistance, and it is important to support its shortening as a valid marker of emerging resistance. Sangster (1999) assumed that shortened ERP would be the first sign of emerging resistance to macrocyclic lactone drugs. Indeed, Lyons et al. (2008) reported IVM failure at 5 weeks post-treatment, half of the original efficacy described to this anthelmintic of 8 to 10 weeks. This study aimed to run a dose titration trial of IVM in naturally infected horses, analyzing the shedding of parasite eggs.

## Materials and Methods

### Titration dose trial

The present work describes a titration dose trial of IVM (Eqvalan 10 mg/mL, Merial, Campinas, Brazil) given orally and used at 100, 200 (label dose), 300, and 400 µg/kg of live body weight.

### Animals and management

All animals were born and raised in a high-performance Thoroughbred stud farm in Paraná, Brazil (25°40’21.3”S 49°12’34.3”W) (Dall’Anese et al., 2023). The climate is subtropical classified as Cfb on Köppen criteria. The annual mean temperature is 17 °C and rainfall is 1,550 mm^3^. The animals were subjected to the rotation of anthelmintics every 60 days, starting at 2 months old with no previous FEC. Fenbendazole, moxidectin, piperazine, an association between ivermectin and praziquantel, or an association of moxidectin and pyrantel were previously administered to all horses as part of the parasite control program.

Healthy (n = 28), 12 to 14 months old, weighing on average 287 kg Thoroughbred yearlings of both sexes (13 fillies and 15 colts) were enrolled in the study. The foals were clinically healthy and fulfilled the inclusion criteria of age, and no anthelminthic treatment 30 days before the trial. The animals were fed twice daily (18% protein) for their age and weight and were kept on pasture (*Cymbopogon* sp.). The animals were randomly assigned to one of four groups with a minimum of 200 FEC individually. All groups had an average FEC of approximately 1000 at the start of the study. All animals were treated with IVM on the same day, after weighting on a digital scale.

### Coproparasitologiacal analysis

Ten grams of feces were collected directly from the rectum, packed in styrofoam boxes, and transported to the laboratory (35 km), where they were analyzed on the same day. Sample collection and analysis were done on day 0 and at 14- and 35-days post-treatment (pt). Parasite eggs were identified and quantified using a modified McMaster flotation technique with a sensitivity of 25 EPG (Dias de Castro et al., 2017). For each group, larval cultures were set up based on mixed fecal samples on days 0 and 35. Third-stage larvae (L3) were identified following Santos et al. (2018).

### Statistical analysis

The efficacy of the titration trial was assessed based on individual pre- and post-treatment FEC. The FECR was calculated using a Bayesian hierarchical model (Torgerson et al., 2014) through Math eggCounts-2.3 for R version 3.6.1 (UZH, 2024). The uncertainty interval (UI), the level of confidence interval at 0.05 (CI/alpha), and the lower confidence interval (LCI), also known as the highest posterior density (HPD) for the data, were also determined. Lastly, the Kappa values were set to determine the function of the Bayesian analysis for data credibility (exploration vs. exploitation). As previously performed by von Samson-Himmelstjerna et al. (2007), the goal of this trial was to determine if at w5pt egg production exceeded 10% of pre-treatment cyathostomin FEC levels (< 90% efficacy). A descriptive analysis was included to compare the FEC data among treatment groups.

## Results

Cyathostomins were the predominant (98%) parasites identified in all larval cultures and were the only ones included in the statistical analysis. *Strongylus* spp. larvae were present in a variable (< 2%) number.

**Figure 1:**
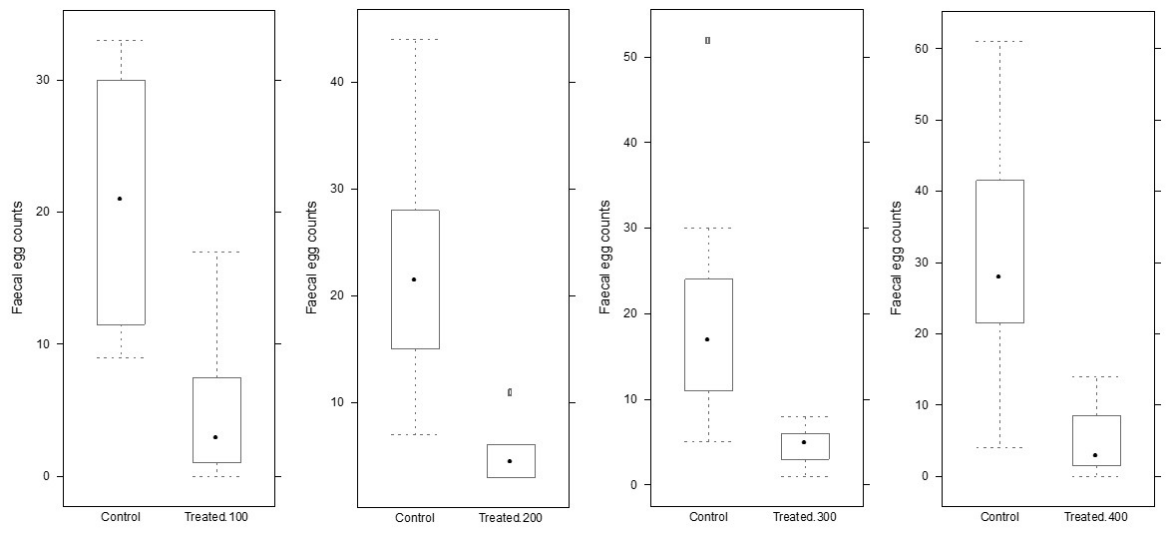
Box-whisker Plot of fecal egg count for the untreated (control/day zero), and treated with ivermectin (100, 200, 300, and 400 µg/kg) at week 5 post-treatment in Thoroughbred yearling horses. Obs. One horse had to be removed from the 200 µg/kg group.

Table 1 demonstrates the complete FEC data of male (M) and female (F) yearlings after IVM treatment. The average FEC before the trial was 1000 (+/- 540). Parasite eggs were not found on w2pt for any tested dose, but FEC reappeared on w5pt (up to 850). We found no sex or weight-related correlation for the ERP (data not shown). Figure 1 shows the descriptive analysis of FEC comparing control (day zero) with the IVM-treated groups on the w5pt using the average of real FEC. Even though there was a statistically significant reduction (P > 0.05) in egg counts at w5pt, we have noticed a wide margin of FEC in animals treated with 100 µg/kg of IVM. The results were not statistically different between the IVM concentrations (P > 0.05).

**Table 1:** Fecal egg count (FEC) of male (M) and female (F) at day zero, week 2 (w2pt), and week 5 (w5pt) after ivermectin treatment in Thoroughbred yearling horses.

Sex	Weight (kg)	Dose (µg/kg)	Day 0	w2pt	w5pt
F	248	100	1650	0	150
F	250	100	550	0	100
M	252	100	450	0	350
F	275	100	600	0	850
M	275	100	1600	0	0
M	296	100	1050	0	400
M	335	100	1400	0	0
F	266	200	750	0	250
M	277	200	1250	0	150
F	280	200	2200	0	150
F	293	200	1400	0	550
M	299	200	900	0	300
F	300	200	350	0	200
M	275	300	2600	0	300
M	290	300	250	0	300
M	292	300	900	0	150
M	321	300	1500	0	50
F	322	300	850	0	400
F	307	300	400	0	150
F	337	300	700	0	250
M	228	400	1700	0	50
M	270	400	200	0	150
M	272	400	1100	0	100
M	281	400	1050	0	0
F	305	400	2450	0	400
F	309	400	3050	0	450
M	312	400	1400	0	700

A series of parameters were estimated by running a Bayesian hierarchical paired model. Table 2 shows the percentage of FEC reduction between the beginning of the experiment and w5pt. The above descriptive values were not statistically different, as IVM at 100 and 400 µg/kg showed a CI above the established threshold, probably because of the sample size. To convey the uncertainty data while running a Bayesian analysis the UI values were preferable using. It is safe to say that UI had a similar interpretation as the CI or even HPD were compatible in our study. The Kappa values (0.5 – 0.6) showed a satisfactory agreement with the categorical coefficients, meaning that our data showed a correct balance representation. It was not possible to establish an LCI for IVM at w5pt as we got full efficacy on w2pt and had no linear trend for FEC on w5pt.

**Table 2:** Bayesian parameters determining the effect of different concentrations (µg/kg) of ivermectin (IVM) on fecal egg count reduction (%), uncertainty intervals (UI), lower confidence interval (LCI)/highest posterior density (HPD), and kappa values between untreated and treated horses five weeks post-treatment (w5pt) in Thoroughbred yearlings.

IVM	w5pt (%)	UI	LCI/HPD	Kappa
100	74.8	0.42-0.96	46.4-98.2	0.6
200	73.7	0.43-0.87	49.3-90.0	0.5
300	71.9	0.42-0.86	46.4-88.1	0.5
400	82.9	0.61-0.92	65.1-94.3	0.5

## Discussion

Although IVM still presents considerable efficacy against cyathostomins worldwide, the present report is of great concern, shedding light on the prevalence and the possibility of having an early indicator of its resistance. All concentrations had 100% efficacy at w2pt but this did not guarantee a high efficacy for much longer. Testing the parasite population at w5pt allowed us to determine the shortening of ERP of IVM at an under-, a therapeutic-, and two high-dose levels. It is worth reporting that a limitation of the study was the analysis performed only on two experimental days post-treatment.

Assuming the resistance status of the present cyathostomin population at w5pt, egg reappearance results from the egg suppression period of IVM from remaining adult parasites. The emergence and development of late L4 in adults must also be considered before reporting drug failure. Multiple drug resistance has been determined for cyathostomins treated with IVM, moxidectin, pyrantel, and fenbendazole from 11 stud farms in four different states in Brazil (Canever et al., 2013). As one of the strict pieces of advice, the authors have recommended banning the use of fenbendazole in those farms due to the considerable drug failure (< 70% efficacy) and the consequent risk of clinical cases. Along with the increase in drug concentration, the short treatment interval used in stud farms may be the two most important risk factors for selecting drug-resistant cyathostomin populations. Once again, the possibility of withdrawing the therapeutic use of IVM when the drug reaches less than 80% FEC reduction at w5pt needs to be considered. Other possibilities for returning drug efficacy could be the use of drug enhancers and MDR modulators with current anthelmintics, maintaining parasite diversity causing a dilution effect (Civitello et al., 2015), the use of bioactive pastures, and other innovative therapies, i.e., Biometals.

Even though the use of target-selective treatment (TST) did not impact the Strongyles community diversity of horses in Sweden (Halvarsson et al., 2024), due to a low external influx of infected animals, this may be a fundamental advantage when considering the genetic dilution effect when looking at the same parasite community but in a different genetic historical context. The continuous use of chemicals during long periods would decrease ecological biodiversity (genetic and phenotypic). Therefore, horse farming would benefit considerably from adopting a low-input farming system (LIFS) supporting the sustainable agriculture concept. Parr et al. (1990) defined LIFS as an on-farm process to reduce external contributions. Therefore, the use of LIFS, associating long-term management strategies, such as the TST, mix crop-livestock (Schafaschek et al., 2021; Babola et al., 2023), and breeding (kinship effect) (Dall’Anese et al., 2023), can create a strong resilient environment, supporting LIFS and horse welfare.

The present data reinforces the idea that using high concentrations of IVM may be a short-lived alternative for parasite control. Instead, disruptive parasite innovations for diagnosis and behavioral indicators for decision-making shall be encouraged. These include the Mini-Flotac (Dias de Castro et al., 2017), nemabiome and diversity index analysis (Halvarsson et al., 2024), and in specific cases the ELISA test (Lightbody et al., 2024). Moreover, a more precise drug failure assessment is necessary to prevent the selection and the spread of anthelmintic resistance populations. Abrahão Pires et al. (2024) suggested that FEC does not correlate well with behavior indicators and should not be used alone as a parasite health management method for horses of any age. Therefore, TST based on FEC and transient threshold abundance (Schafaschek et al., 2021) is recommended to maintain population diversity and low parasite drug selection.

We consider it important to advise that a drug that reaches sub-optimal effect (< 70%) should be restricted from health programs to safeguard horse health and secure welfare. An alternative is to recommend a long drug restriction interval which might allow the dilution of existing anthelmintic resistance by the positive selection of beneficial (signature) alleles to rise (Price et al., 2003; Civitello et al., 2015). However, until today, there is no evidence of the return of high efficacy after such a strategy. As Porter & Crandall (2003) described, reverse evolution is unidentifiable as the ancestor population rate of equilibria is unknown and may have been lost along the way. Another challenge arises in determining the number of mutations that the resistant population would differ from a laboratory or field isolate. Kaltenbach et al. (2015) argued that the new adaptative reorganization may be phenotypically reversible but genotypically irreversible, constituting separate fitness peaks.

Similarly, to the present data, the shortened ERP of the therapeutic dose of IVM and also MOX at the w5pt using the FECRT was reported in horses worldwide. FECRT is a quantitative method that indicates the phenotypic level of efficacy for anthelmintics over a particular population. Environmental factors can also lead to phenotypic selection, driving specific genetic evolution (Price et al., 2003). That’s why a diverse parasite community is essential in maintaining a selective sweep toward the site instead of being suppressed by anthelmintics. As a reminder, we don’t know the level of haplotype(s) related to IVM selection imposing an unknown rate of adaptation or true selection. The rate of hard or soft selective sweep in a population and the production of distinct patterns of genetic variation have been discussed (Harris et al., 2018) in helminth parasites of ruminants and *Drosophila melanogaster* (Redman et al., 2015; Garud et al., 2021) but not for horses. As discussed above, the possible benefits of using more sustainable control strategies would significantly impact the continuity of drug efficacy, securing horse welfare.

## Conclusions

This study documented a 5-week cyathostomin egg reappearance period after IVM treatment in Thoroughbred yearlings, even at 400 µg/kg (double dose).
